# Fiber Reinforced Polymer Strengthening of Structures by Near-Surface Mounting Method

**DOI:** 10.3390/polym8080298

**Published:** 2016-08-11

**Authors:** Azadeh Parvin, Taqiuddin Syed Shah

**Affiliations:** The University of Toledo, Toledo, OH 43606, USA; tsyedsh@rockets.utoledo.edu

**Keywords:** near surface mounted, NSM, flexure, shear, prestressed, masonry, URM, bond, temperature, review

## Abstract

This paper provides a critical review of recent studies on strengthening of reinforced concrete and unreinforced masonry (URM) structures by fiber reinforced polymers (FRP) through near-surface mounting (NSM) method. The use of NSM-FRP has been on the rise, mainly due to composite materials’ high strength and stiffness, non-corrosive nature and ease of installation. Experimental investigations presented in this review have confirmed the benefits associated with NSM-FRP for flexural and shear strengthening of RC and URM structures. The use of prestressing and anchorage systems to further improve NSM-FRP strain utilization and changes in failure modes has also been presented. Bond behavior of NSM-FRP-concrete or masonry interface, which is a key factor in increasing the load capacity of RC and URM structures has been briefly explored. Presented studies related to the effect of temperature on the bond performance of NSM-FRP strengthened systems with various insulations and adhesive types, show better performance than externally bonded reinforcement (EBR) FRP retrofitting. In summary, the presented literature review provides an insight into the ongoing research on the use of NSM-FRP for strengthening of structural members and the trends for future research in this area.

## 1. Introduction

**Figure 1:**
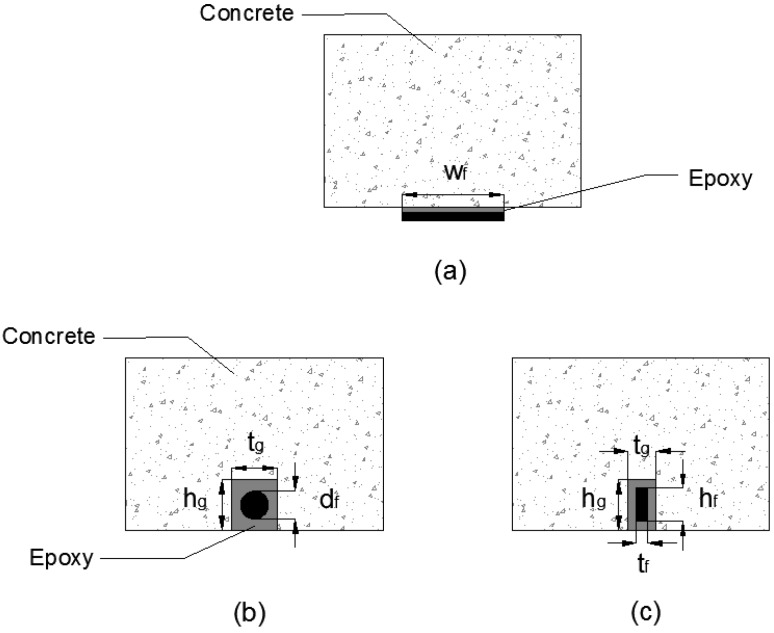
FRP strengthening (**a**) EBR FRP plate or sheet (**b**) NSM FRP rod or bar (**c**) NSM FRP laminate or strip.

Fiber reinforced polymers (FRP) retrofit has been implemented in numerous construction projects worldwide to improve structural performance. Currently, the most common method of retrofitting is the external bonding reinforcement (EBR) method. This method has been employed in practical applications for quite some time and related guidelines have been established in various countries (e.g., ACI 440R 08 [1] and FIB bulletin 40 [2]). On the other hand, near surface mounting (NSM) is a relatively recent retrofit technique that involves cutting out a groove on an existing concrete member, applying an appropriate adhesive and then laying FRP bars rods, or strips into the groove. Figure 1 shows cross-section details of strengthening techniques using both EBR FRP sheets or plates as well as NSM FRP rods and strips.

NSM-FRP is an appealing method to strengthen existing and new structures due to its advantages over EBR, primarily from the improved efficacy achieved from better strain distribution resulting in higher utilization of available strain [3] and its higher resistance to many environmental factors due to being embedded into the concrete cover. Although temperature susceptibility is still moderately high for NSM-FRP, it is considerably reduced by changing the grout filler type. This is a newly emergent research topic area and is still under preliminary investigation [4]. In some cases the FRP bars are covered by the grout so the aesthetic impact of the NSM-FRP method is almost negligible. This is especially important in restoring older monuments and structures that are appraised for their aesthetics. In the case of parking garage slabs with negative moment deficiencies, NSM-FRP can be used to improve their structural capacity since the grooves protect NSM-FRP bars from vehicular impacts. Installation of NSM-FRP is also much easier, as surface treatment of existing concrete surface is not required. Irregularities of the surface or weaker concrete cover, is no longer a concern as the grooves for the NSM-FRP are narrow and deep. Anchorage of FRP bars is easier with the NSM technique, especially for beams with fixed ends where the moments are high. This results in lower premature debonding of FRP reinforcement [5]. Drawbacks of using NSM-FRP, such as lack of protection against vandalism and concrete fracture around NSM laminate grooves can be addressed by using a relatively new method called hybird composite plates (HCPs). These plates consists of strain hardening cementitious composite (SHCC) and FRP sheets, which provide protection against damage and improving transfer of stresses between FRP and concrete [6].

These advantages have been the reason for a sudden increase in the experimental, numerical, and analytical research over the past few years [5]. The main goal of this paper is to provide an overview of ongoing research on NSM-FRP reinforcement applications in the structural engineering field. In the following sections, greater emphasis is placed on more current and emerging topics, such as NSM-FRP applications in shear and masonry design, structural applications using prestressed FRP and temperature related studies. Other areas, such as flexural applications and bond factors are briefly discussed, as they have already been explored in depth in a review by De Lorenzis and Teng [5].

## 2. Flexural Applications of NSM-FRP for Concrete Members

**Figure 2:**
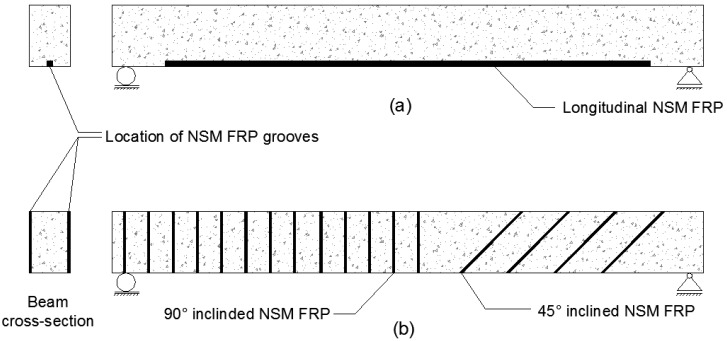
NSM-FRP strengthening of beams (**a**) for flexure (**b**) for shear.

NSM-FRP rods can be a highly effective method to improve the flexural capacity of RC beams. The locations of NSM FRP in both flexural and shear strengthening applications is shown in Figure 2. Table 1 provides a summary of existing main experimental data regarding flexural strengthening applications that are discussed in this section [7,8,9,10,11]. An experimental study performed by De Lorenzis et al. [7] showed that NSM-FRP rods increased the load carrying capacity of RC beams ranging from 25.7% to 44.3%. Test results by Seo et al. [3] also emphasized that NSM reinforcement allowed for more efficient use of FRP reinforcement. Concrete members strengthened through NSM reinforcement had almost 1.5 times higher bond strength as compared to EBR. Even with lower strain distribution, the actual value of strain in NSM-FRP is much higher than EBR, indicating higher material efficiency.

Long term tests over a two-year period performed by Astorga et al. [8] indicated that NSM-CFRP strengthening improved the ultimate load bearing capacity of concrete bridge slabs by 41% and yield load bearing capacity by 20% on average. Although they noticed a decrease in ductility, they also noted that strains were successfully transferred from concrete to NSM-CFRP and allowed for full exploitation of ultimate strain of CFRP.

Barros et al. [9] performed several tests on flexural strengthening capabilities of NSM-CFRP retrofitted RC beams and compared them to EB-CFRP retrofit. They noted that NSM reinforcement was the most effective technique in terms of ultimate load carrying capacity. However, the effectiveness was less profound with the increase of CFRP reinforcement ratio. This reduction in effectiveness is explained by numerical analysis that indicated a decreasing relationship of CFRP effective strain with an increase in the CFRP reinforcement ratio. Experimental tests performed by Al-Mahmood et al. [10] additionally reported that strengthening of RC members using NSM CFRP rod reinforcement provided a change in failure mode from FRP rupture or debonding to compressive concrete crushing. This depended on a coupled ratio between CFRP rod cross-section and CFRP rod length versus concrete compressive strength. The authors mentioned that an accurate threshold to predict the failure mode requires further investigation. Soliman et al. [11] conducted flexural tests on NSM-strengthened RC beams. They observed that along with increase in the flexural capacity, the NSM-FRP system was particularly more effective with RC beams originally having low-steel reinforcement ratio of about 0.4 times the balanced steel reinforcement ratio. A recent study by Baghi et Barros [6] however, showed that effectiveness of NSM and EBR techniques when using higher steel reinforcement ratio can still be maintained using a new technique, called hybrid composite plates (HCPs). This technique employs a thin plate of strain hardening cementitious composite (SHCC) that is reinforced with CFRP sheets and then attached to the concrete surface. Higher stress transfer between the CFRP and concrete subtrate was observed, which subsequently provided improved FRP efficiency.

## 3. Shear Applications of NSM-FRP for Concrete Members

The NSM technique can be used to overcome drawbacks that occur during EB-FRP for shear strengthening due to premature debonding of FRP [12]. Several authors investigated NSM-FRP shear strengthening of concrete members. A summary of testing conditions, specimen details, observed failure modes and test variables of these studies is available in Table 2 [13,14,15,16].

Barros and Dias [13] performed preliminary experiments by developing a NSM shear strengthening technique using CFRP strips. The NSM retrofitting technique significantly improves the load carrying capacity of both unreinforced and reinforced concrete beams. It was also the most effective shear strengthening method of the CFRP systems and provided highest deformation capacity at the failure point of the beam. Similarly, Rizzo and De Lorenzis [14] reported that the use of NSM retrofitting led to a significant increase in load bearing capacity ranging from 22% to 44% as opposed to the control models. In comparison to EBR-FRP, the shear contribution of NSM-FRP was about 2.6 to 2.8 times higher.

Rizzo and De Lorenzis [14] also noted that stiffer and stronger groove-filling epoxy led to a reduction in FRP contribution to the shear capacity. The stiffer bond-slip behavior prompted to higher peak bond stresses and faster debonding crack formation. An epoxy with lower elastic modulus and tensile strength was also tested which showed a more ductile bond slip behavior. This provided lower bond-shear stress, higher failure load carrying capacity and delayed crack initiation.

Increasing the spacing of the NSM bars led to more individual debonding of the bars from the concrete. Closer spacing or higher orientation of the bars led to mutual interaction of adjacent bars causing a debonding failure pattern. This resulted in a lower failure load when compared to the individual debonding of each bar [14,15]. For both spacing and orientation of bars, the failure mode was characterized by the separation of concrete side cover of internal stirrups. This kind of concrete cover failure occurred due to internal steel stirrups creating a vertical plane of weakness separating the steel bars and the concrete close to the NSM-FRP reinforcements [13,14,15]. To overcome this failure, the NSM bars were applied as deep as possible into the beam sections and provided a significant increase in the shear capacity. By increasing the depth of the NSM bars, the concrete failure surface had a higher fracture area, resulting in greater concrete resisting fracture force and shear strengthening effectiveness. Additionally, higher maximum pullout force was attained. Another factor which affects the efficiency of NSM technique for RC beams is the concrete compressive strength. Higher strength concrete led to more efficient use of NSM and changed the failure mode from concrete fracture to debonding of NSM-FRP bars [15].

In order to improve the shear capacity of RC beams, manually made (MM) FRP rods along with an anchorage system were employed. An increase in shear strength of 25% to 30% was reported for unanchored MM FRP and 41% to 48% for anchored MM FRP strengthened beams, over the control model. Using the anchorage systems also led to the beam exhibiting flexural cracks and an increase of 40% to 75% in ductility over unanchored specimens [16].

## 4. Prestressed NSM-FRP Applications for Concrete Members

In order to further improve flexural performance and utilization of FRP strain, prestressing of NSM-CFRP components has been a recent area of study with excellent results [17,18,19,20,21,22,23,24]. A summary of testing conditions, presstressing levels, test variables and observed failure modes of NSM FRP presstressing experiments [17,18,19,20,21,22,23,24] are presented in Table 3.

Hajihashemi et al. [17] tested simple-span RC beams strengthened with NSM CFRP strips that were prestressed to 0%, 5%, 20% and 30% of their ultimate strain. Results indicated a 9% increase in yield load and a 15% increase in ultimate load capacity when using 30% prestressed NSM-CFRP strips. This was achieved from higher tensile strains noted in the NSM-CFRP laminates, which reached their ultimate strain and failed via rupture. A lower final displacement was noted for all prestressed beams as compared to control and nonprestressed ones. Moreover, the crack distribution and behavior of the prestressed NSM-CFRP beams under service loads was improved as the number of cracks were reduced to only 5%–30% of control and unprestressed beams and the crack widths were also reduced to only 22%–52% as well.

Hong and Park [18] A maximum prestressing level of 50% or lower was recommended for NSM-CFRP strengthened RC and prestressed beams based on the load-deflection response, strain development, ductility and energy absorption. The results showed that failure mode of the prestressed specimens changed from premature debonding, as with nonprestressed beams, to concrete cover separation at the end of the NSM-CFRP plate. Improvements of 11%–67% in cracking loads, 40%–77% in yield loads and an average of 85% in ultimate loads was observed, whereas deflection decreased by 43% over the unstrengthened beam. This decrease in deflection ductility is typical with most prestressed NSM-FRP strengthening studies, as the higher strain achieved from prestressed NSM-FRP results in a downward shift of the neutral axis. This provides a greater uncracked zone in the cross section and cracking moment, which causes more flexural rigidity for the prestressed beam [18].

Limited studies has been performed related to fatigue loading of RC beams strengthened using prestressed NSM-FRP. Oudah et al. [19] tested full-scale RC beams, under 3 million cycles of load at a 2.0 Hz frequency to simulate service loading conditions. The beams where strengthened with NSM-CFRP strips with prestressing levels of 0%, 20%, 40% and 60%. During the first 500 cycles of the experiment, rapid increase of strain and crack width were noticed, which then stabilized. Fatiguing of NSM-FRP occurred when the tension steel reached the same minimum and maximum tensile stress values. Higher prestressing levels led to quicker stiffness reductions due to increasing concrete cyclic creep stress. Debonding resistance at the epoxy-concrete interface was improved as prestressing amount was increased. Crack widths at the end of the cycle were still lower than the Canadian standard association (CSA) maximum allowed. There was a need to develop an alternate anchorage system for fatigue loading due to the possibility of FRP slippage at end anchorage at higher prestressing levels [19].

In addition, RC slabs can also benefit from using prestressed NSM-CFRP. Hosseini et al. [20] tested three 2400 mm long, regular 120 × 660 mm^2^ cross-section RC slabs with 0%, 20% and 40% levels of prestressing, under a four point bending load. The test results indicated that prestressing NSM-FRP laminates for strengthening of RC slabs is a highly effective method for improving flexural performance. With just 20% prestressing level of NSM-CFRP, a 55% improvement in service load and a 136% in ultimate load capacity were achieved. The 40% level of prestressing guaranteed a 119% improvement in service load and 152% improvement in ultimate load carrying capacity. As with RC beams, RC slabs also showed a lowered displacement at maximum load but the deflection ductility remained reasonably high (over a value of 2.0).

The effectiveness of strengthening low concrete strength (15 MPa) RC slabs with prestressed NSM-CFRP was also explored by Hosseini et al. [21]. Just like their earlier regular concrete strength RC slabs test, three of the slabs were strengthened with NSM-CFRP of 0%, 20% and 40% levels of prestressing and their behavior, failure modes and FRP performance were investigated. With only 20% prestressing level the cracking, service and ultimate loads were increased by 258%, 123%, 125%, respectively. When using the 40% prestressed NSM-CFRP, a guaranteed improvement of 40% for yield, 190% for service load and 134% for ultimate load was attained. Despite lower overall maximum deflection, the deflection ductility was around 1.9, indicating adequate performance for RC slabs.

For all tested prestressed NSM FRP RC slabs (normal and low concrete strength) by Hosseini et al. [20,21], failure mode was the rupture of NSM FRP laminates, which is more favorable than FRP debonding failure. This was an indication that the FRP material was highly effective. In comparison with normal strength concrete of 46.7 MPa, low strength concrete of 15 MPa with prestressed NSM-FRP showed better performance and effectiveness at serviceability limit state conditions. A lower load carrying capacity and FRP effectiveness was noted for higher steel reinforcement ratio [21]. To further understand this behavior, Rezazadeh et al. [22] conducted a study to explore the benefits of using lower steel reinforcement ratio of RC beams strengthened with prestresssed NSM-CFRP laminates. Five beams were designed with steel reinforcement ratios of 0.39% which were low enough to fail the deflection limit of the serviceability limit state conditions. NSM-CFRP laminates with prestressing amounts of 20%, 30% and 40% of nominal tensile strength were used to strengthen the beams. Results of the experiment indicated that both cracking and yield load improved. This was due to an initial compressive strain in the tensile steel and surrounding concrete, due to the applied prestressing force of the NSM-FRP. Service load capacity increased significantly for the prestressed FRP beams as compared to non-prestressed FRP beams, failure mode changed from concrete crushing to FRP rupture by prestressing. In order to maintain the ductile behavior of RC beams, an upper prestressing limit is necessary due to the reduction in the ultimate deflection.

Stone slabs with no tensile reinforcement were investigated by Ye et al. [23]. The tested stone slabs were around 3200–3400 mm long with a 100 × 450 mm^2^ cross-section area and had a compressive strength of 142.4 MPa. The slabs were strengthened using NSM-CFRP bars prestressed to 0%, 15%, 30% of their ultimate strength. The results showed significant improvement in the behavior of the slabs. The 30% prestressed NSM-CFRP specimen provided a 60% improvement in cracking load and the width of the cracks were also reduced significantly. Furthermore, the failure mode of strengthened specimens was changed to a more ductile flexural failure, instead of abrupt fracture failure which is common with unstrengthened stone slabs. Compared to the non-prestressed FRP specimen, prestressing provided much higher FRP strain utilization.

To reduce or delay the bond failure associated with prestressing NSM-FRP bars, more emphasis on anchorage systems and end treatments is required. To investigate these, Wu et al. [24] tested concrete beams with appropriate strengthening and anchorage systems. A 40% prestressing level for the NSM-CFRP bars was selected and three different end treatments were tested: (1) mechanical U-shaped steel-plate hoops; (2) expanded grooves at the ends of the bars; (3) as well as use of a lower modulus resin at the ends of the bars. As expected, prestressing NSM-CFRP bars improved the flexural capacity and led to reduction in deflection ductility of the beams. Using two FRP bars instead of one resulted in a 50% reduction of displacement ductility and a 60% reduction in energy ductility index. Similar to previous studies results, ductility was reduced and higher efficieny of FRP was noted. The end treatment tests were intended to reduce the end debonding failure that occurs frequently with NSM strengthening. It was noted that the simple mechanical U-shaped steel-plate hoops proved most effective end treatment and led to concrete crushing failure and higher ductility index, with no effect on yielding or ultimate load capacities. The lowered modulus epoxy specimens failed via premature end concrete splitting failure and did not provide satisfactory end treatment performance.

## 5. NSM-FRP to Concrete Bond Performance

The bond behavior of the NSM reinforcing is of critical importance in determining the effectiveness of NSM technique [5]. The strength of the NSM technique is dependent on a number of factors which control the bond-slip behavior, such as, surface treatment of the FRP bars or rods, type of adhesive, groove and FRP dimensions [25,26]. As previously mentioned, bond performance factors have been briefly investigated in this paper. More detailed review can be found in De Lorenzis and Teng [5].

### 5.1. Surface Treatment of FRP Bars or Rods

In general, surface properties of NSM reinforcements provide frictional forces and mechanical interlocking which affect the bond behavior. According to bond tests performed by Lee et al. [27], from the highest to lowest in terms of strain achieved from surface treatments are sand coated, ribbed, roughened, spirally sand coated and smooth. Each type of surface treatment accounted for various levels of damage to concrete and epoxy and there are at least nine different failure modes, including a combination of cracking, splitting and FRP breakage.

### 5.2. Adhesive Types

The influence of adhesive type on the bond behavior and failure load capacity for flexural reinforcement is quite significant. Sharaky et al. [28] conducted tests using resin and hardener based epoxy with higher ductility and noticed improved load capacity and stress distribution along the bond length. This prevented sudden failure and increased the efficiency of having longer bond length. In one case, the failure load was increased by 22.57% but more importantly, the failure mode was changed from sudden FRP slippage to a more ductile and gradual FRP slippage failure. Earlier studies suggest that use of cement based epoxies is limited due to their significantly lower tensile strengths [6].

Soliman et al. [29] tested a fast curing mix of resin plus cement based epoxy which was capable of resisting a high range of temperature and compared it to a high strength resin epoxy. The cementitious based epoxy showed a failure load of only 40%–56% of that of the high strength epoxy due to concrete and adhesive splitting. The high strength resin epoxy showed failure due to concrete tension failure and in case of higher bond length, led to FRP rupture. The main reasons to use cementitious based epoxy are for temperature based advantages. In the case of shear strengthening using NSM bars, tests performed by Rizzo and De Lorenzis [14] revealed that the epoxy with lower elastic modulus and tensile strength led to a significantly higher failure load and showed twice as much FRP contribution compared to high strength epoxy; using lower strength epoxy resulted in a weaker but more compliant and ductile bond slip behavior, leading to stress distribution over a longer length of the FRP bar. This lowered the bond shear stress, delayed crack initiation and debonding failure.

### 5.3. NSM Groove and Bar Dimensions

Increasing the dimensions of the groove generally led to a slight increase in bond strength between NSM-FRP bars and concrete but had no effect on the failure mode [27,28]. When using cement based epoxies, a larger groove width provided a slightly higher bond strength as compared to resin based epoxies [28]. However, increasing the groove size can sometimes result in reduced bond strength due to the shrinkage of cement in bigger grooves. Thus a groove width greater than 1.5 times NSM-FRP bar diameter for cement based epoxies is not recommended [29]. Increasing the diameter of NSM-CFRP bars from 8 to 9 mm resulted in an improvement of 21.95% in the load capacity. In the case of GFRP bars, increasing the diameter from 8 to 12 mm resulted in 72% increase in load carrying capacity. The failure mode remained the same for both cases [28].

### 5.4. Bonded Length

The pullout tests by Soliman et al. [10,29] on NSM-FRP systems using FRP bars and epoxy had also confirmed that increasing the bonded length of NSM reinforcement resulted in higher stress distribution along the length and reduced the bond stress. This led to higher load carrying capacity which was limited up to an approximate length of 48 times the diameter of the NSM bar. Pullout tests by Sharaky et al. [28] using NSM-CFRP bars similarly revealed that increasing the bonded length by 25% provided a 17.2% improvement in failure load. Accordingly, it was understood that bond length was one of the main factors influencing the bond failure.

## 6. NSM-FRP Applications for Masonry Walls

Due to the susceptibility to seismic impacts and brittle failure mechanisms, URM walls can benefit from NSM retrofitting as it is a cost effective and minimally-invasive retrofit technique. NSM bars achieve higher axial strains, along with previously mentioned benefits over EBR applications, resulting in improved flexural and shear capacities, as well as overall ductility. Similar to other structural applications, the study of FRP bond interface behavior of masonry walls retrofitted with NSM-FRP is of critical importance.

### 6.1. NSM-FRP Masonry Bond Studies

Perterson et al. [30] conducted pull tests to investigate the bond behavior of solid clay brick masonry prisms strengthened with both vertically (CFRP strips perpendicular to bed joints) and horizontally (CFRP strips parallel to bed joints) aligned NSM-CFRP strips. The selected NSM-CFRP strips were of rectangular shape to maximize the confinement from surrounding concrete. Tests showed that debonding of FRP from masonry was the main failure mode for both orientations of CFRP strips. For prisms with horizontally aligned CFRP strips, a compression load to simulate a vertical compression in masonry structures was applied. In the absence of vertical compression load, pull-through failure was observed at lower loads. An 8% decrease in the bond strength was noticed when vertical NSM-CFRP strips were used. If the vertical NSM-CFRP strips passed through a head joint, the bond strength was further reduced to 11%. A larger decrease (31%) in bond strength was noted in the case of horizontal FRP strips when the distance between FRP and the mortar joint was the least. In general, NSM CFRP strips oriented parallel (horizontally) to mortar joints showed significant reductions in bond strength unless a compressive load of 1.0 MPa was applied. The application of the compressive load allowed for the bond strength of the horizontal CFRP strips to perform similar to that of the vertical CFRP strips. It was also noted that deeper NSM grooves led to cracking of the prisms. This indicated that unless using a shorter groove depth, NSM-FRP retrofitting might be adversely affected by this cracking failure, especially for weak, hollow or cored masonry. In comparison to EBR-FRP, the percentage reduction in bond strength was lower and associated failure modes were broadly different than retrofitting with NSM-FRP strips of both horizontal and vertical orientations.

Willis et al. [31] also conducted an extensive study to understand the influence of various parameters such as surface preparation, geometric properties, bonding agent of bed joints and the location of FRP relative to core and perpend joints. A series of pull tests were conducted using both EBR-CFRP and NSM-CFRP to retrofit unreinforced modern clay brick masonry walls. The results showed that for NSM-CFRP, higher bond strength can be achieved by increasing the embedment of the strip in the groove. The embedment depth cannot exceed the depth of the brick core as it leads to lower confinement from surrounding concrete. Also, placing of NSM-FRP strips through the perpend joints resulted in a 10% reduction of bond strength and is not recommended. The bond slip curves indicated that NSM-CFRP provided twice as much shear stress performance when compared to EBR-CFRP, along with significantly higher bond strength and ductility.

To study the effect of cyclic loading on debonding resistance of NSM-CFRP strips to masonry joints, Kayashap et al. [32] conducting bond tests. The results indicated that NSM-FRP embedment depth was more effective than FRP width at improving bond strength or debonding resistance. This was mainly due to an increase in more efficient confinement from surrounding concrete. All specimens failed via intermediate crack (IC) debonding failure. The authors reported somewhat inconclusive results when trying to study the effects of cyclic loading and hence urged for further research in this area. A database of FRP to masonry pull tests including, the effect of FRP dimensions, concrete strength and the maximum shear stresses and slip results reported by researchers in the last decade was compiled. They also presented 15 existing concrete-masonry bond strength models for comparison purposes. A new analytical model applicable to both EBR and NSM techniques was derived. The analytical model took into account the geometric variables such as FRP strip aspect ratio, material design variables such as axial rigidity of the FRP strip and masonry unit tensile strength.

### 6.2. In Plane Shear

**Figure 3:**
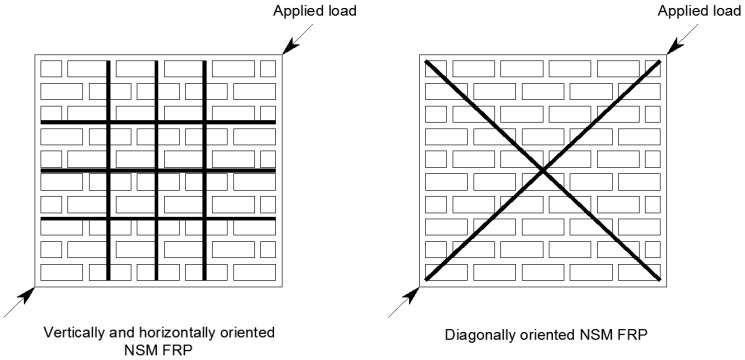
Diagonal shear test of masonry walls retrofitted with NSM FRP.

Figure 3 displays examples of NSM-FRP strengthening orientations for in plane shear applications. The test variables, specimen details and observed failure modes of masonry related in plane shear existing experimental studies in the literature are summarized in Table 4 [33,34,35,36,37]. In plane shear strength of URM walls can be improved using NSM-CRFP strips. Compared to as-built masonry wall, an increase in the maximum shear strength ranging from 1.3 to 2.6 times for retrofitted walls and from 1.3 to 3.7 times for repaired walls was observed [33]. Ductility was also improved substantially, ranging from 2.6 times for walls retrofitted on one side to 25.5 times for the walls with retrofit on both sides and from 2.5 to 10 times for repaired walls. In tests using diagonally oriented CFRP strips, higher axial strain was noted due to high compressive loads normal to the strips. In general, it was noted that the higher ratio of vertical NSM-CFRP strips led to a linear increase in wall panel shear strength.

Similar results were observed in an experimental study performed by Petersen et al. [34]. URM walls were tested in diagonal tension/shear according to ASTM E519-93 and were strengthened using either vertical, horizontal, or a combination of NSM-CFRP strips. The use of vertical NSM-FRP strips resulted in a 28% increase in load capacity when the strips were applied only to one side and a 46% increase when applied to both front and back sides of the URM walls. The vertical strips also proved to be effective in preventing sliding URM failure and showed higher tensile strain, indicating that the strips resisted opening of the sliding cracks. When both horizontal and vertical NSM strips were employed, the horizontal strips prevented opening of diagonal cracks and the vertical strips prevented sliding failure. A 32% increase in loading capacity was also observed.

Mahmood et al. [35] conducted diagonal compression tests on solid clay brick masonry wallettes using a combination of various FRP strengthening techniques including NSM-CFRP pultruded rectangular bars and EB-CFRP/GFRP plates. A 31% to 325% increase in shear strength using FRP over un-strengthened wallettes was observed. The use of NSM-CFRP bars on only a single side of the wallettes proved ineffective. Shear strength and ductility were improved when both sides of the wallettes were strengthened using the NSM technique, with diagonal shear cracking of in-plane walls being the failure mode. Horizontally oriented FRP failed from sliding of the wall along the bed joint (sliding shear failure mode) of the URM but vertical or diagonal FRP was able to prevent this kind of failure. Pseudo-ductility was also enhanced for the specimens that failed via diagonal shear cracking failure. Overall, the GFRP-strengthened specimens showed lower cases of debonding failure than their counterpart CFRP-strengthened specimens.

Konthensingha et al. [36] tested the effectiveness of URM shear panels, which were previously damaged when subjected to compression and cyclic shear loads at three different levels. The walls were then repaired and strengthened with vertical or horizontal NSM-CFRP strips, vertically pre-compressed to 1.4, 2.0 and 2.8 MPa combined with an increasing in-plane cyclic lateral displacement. The results showed that NSM-CFRP was capable of restoring the maximum load capacity of the repaired walls back to pre-damaged URM conditions and provided higher displacement capacity at the same load level. The URM shear panels strengthened with combined vertical and horizontal NSM-CFRP strips, showed improved performance in terms of load, displacement and energy dissipation capacities. At higher pre-compression stress of 2.8 MPa, the pre-existing damage did not seem to affect FRP strengthening effectiveness. Additionally, the energy dissipation was improved.

The same researchers [37] also tested fully fixed (zero rotation) support conditioned wall panels with two different dimensional aspect ratio of 0.5 and 1, that were also vertically pre-compressed and then subjected to monotonic or in-plane cyclic lateral displacement. Some of the walls were strengthened using either horizontal, vertical, or a combination of both NSM-CFRP strips. It was noted that walls with an aspect ratio of 1 showed diagonal shear cracking failures, while the walls with an aspect ratio of 0.5 failed mainly due to sliding failure of the base. Results showed 133%, 382% and 108% increase in displacement capacity, energy dissipation and ductility respectively, as compared to un-strengthened walls. Vertical or vertical and horizontal combination for the orientation of NSM-CFRP strips led to a 9% increasing in loading capacity, whereas horizontally oriented FRP did not show improvement in load capacity but, provided much larger displacement capacity at the ultimate load.

### 6.3. Out of Plane Flexure

Historic clay URM structures are also known to lack out of plane flexural capacity and are susceptible to one-way or two-way bending loads, which can drastically reduce their load carrying capacity. NSM retrofitting in this case is a very viable option due to the low impact on aesthetics, ease of installation and practicality. Several authors studied out of plane flexure tests on masonry structural members. The test variables, specimen details and observed failure modes of these investigations are summarized in Table 5 [38,39,40,41].

Griffith et al. [38] tested 15 NSM-FRP strengthened masonry walls and noted that the spacing of NSM-FRP strips played an important role in increasing the out of plane flexural bending capacity. In one wall with three FRP strips and spacing of 357 mm, the vertical one-way bending capacity improved up to 20 times when compared to the control wall. Displacement was also increased by more than 60%. Higher NSM-FRP reinforcement ratio resulted in an increase in the strength and a reduction in the displacement. The failure load was directly related to the perimeter of the debonded failure plane. In calculating the perimeter, the failure surface which occurs in the brick unit and not at the FRP-adhesive interface, was considered. Closer spacing with smaller FRP strips led to an improvement in the load capacity as long as the FRP strips did not rupture prematurely. Dymtro et al. [39] also performed out of plane shear tests on clay URM walls retrofitted vertically with NSM-CFRP strips. They reported significant improvement of about 3.05 to 6.21 times in flexural strength as compared to the as-built URM wall. In all tested specimens, the ductility was also improved.

Another study by Korany et al. [40] involved developing a minimally invasive retrofitting technique using a combination of drilling holes through URM bricks or the surface of façade walls. Flexible CFRP cables were installed at bed and head joint locations using the NSM technique. To test this design setup and investigate various parameters such as support conditions, axial load or precompression value and reinforcement ratio, ten full size walls were retrofitted with the FRP design. They were tested by applying monotonically increasing uniform out of plane lateral pressure. The results showed that even very low FRP reinforcement ratios of 0.006% and 0.009% using vertically and horizontally aligned FRP respectively, resulted in a 25% increase in load capacity with a 200% to 400% increase in energy absorption and high deformation capacity [40]. Increasing FRP reinforcement to 0.009% vertically and 0.0135% horizontally resulted in a 58% increase in load capacity, 291% increase in energy absorption and 41% increase in deformation capacity. A low precompression value of 0.20 MPa or a 50% increase in FRP reinforcement provided similar improvement in out of plane resistance. Precompression also provided significant enhancement to cracking and ultimate loads of URM and retrofitted walls. Higher pre-compression values in nonbearing URM walls did not provide improvement in out of plane resistance but still showed a 200% increase in energy absorption and a significant increase in deformability. In terms of support conditions, as compared to rigid floor supports, flexible floor supports provided 50% lower load and energy absorption capacities. Flexible floor support specimens failed in compression via masonry crushing, while rigid floor supports failed due to FRP rupture.

NSM-CFRP strips or NSM twister steel bars were also used to retrofit historic URM walls. Field test were performed on five one-way vertically spanning solid walls and four two-way spanning partition walls [41]. These walls were constructed using clay brick masonry. In-situ static out of plane loadings were then applied. Results showed that in one-way spanning walls, post-cracking out of plane flexural strength was increased significantly, even with a single NSM CFRP strip. These increases in wall strength ranged from 440% to 830% for most walls and also provided improved residual displacement capacity ranging from 20% to 35%. It was noted that NSM-CFRP strip debonding failure was associated with providing higher ductility, whereas NSM-CFRP strip pull-out failure indicated brittle behavior.

For the pre-cracked two-way spanning walls, it was noted that retrofitting led to stiffness improvement by 67% compared to the as-built wall. However, due to the historic nature of the case study variables such as prior boundary conditions, material properties and loading history were unknown and thus effectiveness of the FRP strengthening could not fully be investigated. For the non-cracked two-way spanning walls, out of plane strength improvements were much higher than the calculated values in comparison to non-cracked one-way spanning walls. The non-cracked two-way walls also showed a reduction in out of plane stiffness due to either, the top edge of the wall being cracked or unrestrained. Overall, even the weaker pre-cracked walls strengthened with NSM-CFRP strips were enough to satisfy current seismic load standards [41].

## 7. NSM-FRP Applications under Temperature

Apart from the mechanical strengthening advantage, the use of NSM-FRP over EB-FRP reinforcement can provide protection from environmental and fire damage due to additional embedment from concrete cover [42]. In this section, several existing studies in the literature related to NSM-FRP reinforced members subjected to temperature are discussed. Main test variables, specimen conditions, insulation types and failure modes, of these investigations are presented in Table 6 [43,44,45,46,47,48,49,50,51,52]. Yu and Kodur [43] revealed that the NSM-CFRP strips and bars themselves provided better resistance to high temperatures in comparison to external FRP laminates and internal FRP reinforcement.

The effect of the temperature ranging from 20 to 600 °C, on the tensile strength and elastic modulus of NSM-CFRP reinforcing strips and rods was tested [43]. The results showed three stages of tensile strength degradation. In the first stage, the temperature ranged from 20 to 200 °C and the CFRP tensile strength degraded slowly to about 80% of that at room temperature. In the second stage of temperature ranging from 200 to 400 °C, the degradation of tensile strength was rapid mainly due to decomposition of polymer resins at 300 °C. By this stage the tensile strength was reduced to 50%. In the third stage, when the temperature varied between 400 and 600 °C, the degradation of tensile strength was also fast and down to 10% of that of the original. The effect of the temperature on elastic modulus followed a similar trend but was more directly based on the decomposition of the polymer resin. It was also noted that with the increase in temperature, the ultimate strain of CFRP reduced leading to a reduction in ductility.

However, the guidelines for the design of FRP-strengthened structures state that the strength of FRP is to be ignored unless a fire protection system or insulation is in place [1,2]. This is done in order to maintain the FRP system under the lowest critical temperature, which is mainly the glass transition temperature (*T*_g_) of the epoxy adhesive [44,45]. Burke et al. [4] conducted experiments to study various factors affecting the performance of insulated NSM-strengthened RC beams at elevated temperature and reported that adhesive type can have a significant effect on the load carrying capacity. The use of cementitious grout in place of regular resin epoxy was able to provide better durability over four h at 100 °C and over 70 min at 200 °C. The observed failure was the pull out of NSM-FRP strips from the grout, similar to that of ambient temperature. It was also observed that it is not necessary to insulate the full length of NSM-FRP in order to provide fire protection. Insulation of only short length of NSM-FRP strips may be sufficient to maintain effectiveness of bond-critical FRP-strengthening systems.

In another investigation [44], RC beams were capable of achieving two-hour fire endurance rating, even after adhesive temperature exceeded the glass transition temperature, as long as they were well insulated. It was also noted that the residual strength of FRP-strengthened RC beams was equivalent to the flexural strength of the control beam as long as the temperature was maintained below 140 °C for concrete and 570 °C for steel. Palmieri et al. [45] then conducted one-hour fire tests and observed that despite adding higher service load, the NSM-FRP strengthening showed no obvious reduction of strength. The insulation systems could maintain low temperatures of 130 °C for epoxy and 167 °C for mortar. The insulated strengthened RC beam was able to achieve residual strength of 77% as compared to its counterpart at room temperature and, 127% as compared to the unstrengthened beam.

Another extensive study by Zhu et al. [46] was conducted to test the thermal performance of NSM Basalt FRP (BFRP) bars and RC strengthened beams with NSM BFRP bars. According to the authors, the *T*_g_ of resin matrix used during the pultrusion curing process of BFRP bars had higher temperature resistance when compared to CFRP bars. Using the NSM BFRP bars to strengthen beams provided the same amount of fire resistance as the control RC beam. Another point observed, which is in line with the results from Burke et al. [4], is that only partial insulation of the NSM FRP bars was required for fire protection. A 25 mm thick calcium silicate board was required to achieve the two-hour fire endurance when using the NSM high-*T*_g_ BFRP strengthening system.

RC slabs strips strengthened with anchored EBR CFRP sheets and NSM CFRP bars and then insulated with various fire protection systems were studied by Lopez et al. [47]. Calcium silicate (CS) boards or vermiculite/perlite (VP) cement based mortar was applied to the bottom surface of the slabs. The main aim was to keep the CFRP material under its critical temperature (500 °C) and the epoxy/adhesive under its *T*_g_ (glass transition temperature of 55 °C). The bottom surface of the slab was thermally loaded to the ISO 834 fire curve. Insulation with various thicknesses was applied to the anchorage and current (mid-span) zones, while the top surface was kept at ambient temperature to simulate the behavior of one-way slabs in buildings. According to the authors, the NSM-FRP strengthened slabs provided up to 2% better thermal protection using 2 cm of anchorage insulation, 31% using 6 cm and 45% using 10 cm, than their counterpart EBR-FRP strengthened slab. Another observation was that just 2 cm of current (mid-span) zone insulation thickness was enough to provide thermal protection of 90 min for both adhesive and cement based techniques [48].

Similarly, Firmo and Correia [48] applied both service and thermal ISO 834 fire loads on anchored NSM-CFRP RC beams using conventional epoxy and a mixed grout using epoxy and cement binders. Calcium silicate (CS) boards of 25 and 50 mm at the anchorage zones and 25 mm CS boards at the central zone of the beams were used. Results showed the uninsulated control beam lost its CFRP flexural contribution in just 18 min using epoxy and 17 min using the cement binder based grout. Insulating only the anchorage zone with a 25 mm CS board, the epoxy specimen improved the CFRP flexural contribution from 18 to 34 min. Despite highly damaged CFRP bars, a cable mechanism was formed which helped to sustain structural effectiveness. Applying a 50 mm CS board at the anchorage zone and 25 mm CS board at the central zone simultaneously resulted in 114 min long mechanical performance with the average epoxy temperature of 2.2–6.6 times *T*_g_. For EBR specimens provided average epoxy temperature ranging from only 1.2–1.5 times *T*_g_ and CFRP mechanical contribution was lost after 50 min. The authors also suggested that for insulated NSM-CFRP strengthened RC beams, a critical temperature for epoxy can be 2 times *T*_g_.

In a later study, Yu and Kodur [49] also tested NSM-CFRP strips T-beams insulated with Tyfo^®^ CFP insulation systems under ASTM E119 standard fire. Their objective was to study the effect of fire exposure on the specimens, while applying structural loading and different axial restraining conditions. In this study, only the non-anchored region of the beam was exposed to the fire to simulate thicker insulation of the anchorage zones of the FRP. The results show that even for an uninsulated beam, at higher temperatures, a cable action was formed by the CFRP fibers despite burned out CFRP strips at mid-span. This was attributed to the anchorage zone being intact. Furthermore, as long as the anchorage zone for NSM-FRP is insulated, the NSM-FRP system can provide tensile strength even when the rest of the beam is not protected. The insulated beams were able to sustain a 65% of their residual load for 3 h under fire exposure. Axial restraining enhanced the fire resistance of the insulated beams as well. Thus the boundary and loading conditions need to be considered for thermal analysis of NSM-FRP RC beams.

Firmo et al. [50] conducted another study, this time involving double lab shear tests between concrete blocks and NSM-CFRP strips using two adhesive types. The first being a regular epoxy mortar and the second was a mixed grout (MG) using cement and epoxy binders, to investigate the bond behavior at temperatures ranging from 20 to 150 °C. The specimens were heated up to the selected temperatures and then loaded to failure. The results of the tests showed that, bond strength using the mixed grout (MG) reduced by 82% even at 55 °C, whereas the use of epoxy reduced the bond strength by only 16% at 55 °C. Bond slip curves showed MG provided lower stiffness of 10 MPa at 55 °C compared to 16 MPa at 55 °C for epoxy. The failure mode of epoxy changed from concrete shear failure to adhesive failure only at elevated temperatures. The MG always failed from adhesive failure at all temperatures. Overall, mixed grout specimens despite containing cement binders suffered higher performance reductions than conventional epoxy. Additionally, NSM-FRP specimens showed 1.9 to 2.8 times better bond strength performance when compared to EBR-FRP specimens.

The benefits of using a CFRP material with high glass transition temperature (*T*_g_) of 220 °C and higher decomposition temperature (*T*_d_) of 360 °C, bonded with a cementitious adhesive via the NSM strengthening method was also investigated [51]. Dynamic mechanical analysis (DMA) and thermogravimetric analysis (TGA) were performed on the CFRP and thermal conductivity tests on the cementitious grout, to confirm their *T*_g_ and *T*_d_ values. Follow up tests on end anchored NSM-CFRP strengthened RC beams under fire exposure were conducted. Results showed that beams that were heated at the mid-span did not fail even after 90 min of heating. At that point, the CFRP bar indicated a temperature of 600 °C and was completely debonded. This was due to failure of the adhesive interface at elevated temperature but the end zone anchorage was cool enough to provide adequate strength and transferred the stresses.

## 8. Emerging and Innovative Methods to Improve the Performance of NSM-FRP Strengthening

An innovative NSM-FRP shape to further improve the performance of NSM and EBR FRP strengthening systems was tested [52]. The authors developed T-NSM-CFRP, which employs a T-shaped CFRP laminate. The aim behind this method was to combine the advantages of both EBR and NSM strengthening, since the T-shape provided 1.5–4.4 times higher cross-section and bonded areas than the typical NSM and EBR CFRP strips. Seven full-scale simply-supported beams were strengthened with the T-NSM-CFRP strips and subjected to six-point bending load. Increases in load capacities up to 250% compared to unstrengthened beams were achieved. The failure modes reported ranged from intermediate crack (IC) debonding accompanied with concrete cover splitting, or IC debonding with CFRP rupture. However, the benefit of using the T-NSM was that as compared to NSM-FRP, higher overall average strains were observed, indicating greater FRP efficiency. Additionally, due to higher available cross-section of the T-NSM, only a quarter of the installation time, labor and cost were required to achieve similar strengthening when compared to NSM embedded strips.

A hybrid bonding method which incorporated the use of EBR steel plate bonded at the bottom face of beams to cover the NSM bars was investigated by Rahman et al. [53]. The objective was also to improve the bond performance between concrete and applied strengthening NSM bars. The test variables of the study were the number and size of NSM FRP rods and the steel plate thickness. According to the results, the hybrid method was successful in preventing premature debonding failure of NSM FRP under monotonic load, as all the strengthened beams failed via flexural shear failure. This method is especially effective in the case of shear strengthening applications, where ACI 440 [1] requirements of edge clearance and spacing between adjacent NSM grooves for preventing premature debonding failures can limit the NSM strengthening applicability. Additionally, the ductility of the strengthened beam was reported to be similar to that of the unstrengthened beam.

Another hybrid technique proposed by Rezazadeh et al. [54] involved the combination of both NSM and embedment through section (ETS) methods, using CFRP laminates. Flexure and shear applications of this hybrid combination were assessed by strengthening four T beams and under four-point bending load. The results indicated that using this hybrid combination of both NSM and ETS methods resulted in higher resistance to debonding failures such as concrete cover splitting and delamination.

The combination of using NSM and ETS methods together was also tested by Kaya et al. [55] to strengthen the support regions of beam-column joints under reversed cyclic load conditions. The beams and columns were strengthend with NSM-CFRP ropes which were anchored using either the NSM or ETS technique. Results indicated that for the ETS technique, a smaller anchorage length provided higher flexural capacity. If the length was increased, the application of epoxy became more difficult, and hence a lower bond strength was noticed. An anchorage length of 250 mm was recommended while using the ETS technique. However, the load carrying capacity was improved at higher drift level when the NSM technique was used to provide anchorage. It was also noted that in the case of retrofitted specimens subjected to cyclic loading, the ACI 440 [1] code overestimated the flexural loading capacity, and hence more conservative cyclic loading efficiency factors for both NSM and ETS techniques were provided.

Using hybrid composite plates (HCPs) technique, which employs a thin plate of strain hardening cementitious composite (SHCC) that is reinforced with CFRP laminates or sheets, has also been shown to further enhance the transfer of stresses between NSM FRP and concrete. The HCPs lower the chances of concrete fracture failure around NSM grooves, as well as improving FRP efficiency. Additionally, they overcome the limitations to FRP efficiency observed when using higher steel reinforcement ratios [6].

## 9. Conclusions

NSM-FRP strengthening is capable of achieving higher strains than EBR, resulting in more efficient use of the FRP material, thus improving flexural and shear capacities and ductility of RC beams. NSM-FRP reinforcements are also more effective when used in RC beams with lower steel reinforcing ratios. Different surface treatments can lead to various degrees of concrete and epoxy damage and FRP strain utilization; specifically, sand coated and ribbed FRP bars provided the highest strain utilization. Use of a more ductile adhesive resulted in improved stress distribution along the bond length. Higher bonded length in NSM-FRP systems generally led to higher stress distribution and improved performance, up to a limit related to the diameter of FRP bars. Groove dimensions in general have a relatively low impact on increasing failure load capacity.

Premature debonding, shown in EBR-FRP strengthened structures under flexural and shear loading, can be avoided by using NSM-FRP. For shear strengthening, NSM-FRP bars could be used along with high strength concrete and a lower strength epoxy for more compliant and ductile bond slip behavior. This could change the failure mode from concrete cover spalling to debonding of FRP and help shear stress transfer between cracks and increase in load capacity. To further improve structural performance of RC structures, prestressing of NSM-FRP is an excellent option in many cases. Prestressing allows for complete utilization of FRP available strain and can change the failure mode from NSM debonding failure to FRP rupture at a much higher load. Consideration needs to be made to ensure the RC structure still maintains ductile behavior when using prestressed NSM-FRP, by limiting prestress levels to below 50% of their ultimate FRP strain.

NSM-FRP could significantly improve in plane shear and out of plane flexural capacities and ductility of URM walls. Tests on NSM-FRP and masonry bond behavior indicated that the location of retrofitting and embedment depth of FRP components play vital roles on bond strength and failure mode. However, a limit to the embedment depth is suggested as it could lead to cracking of the masonry prisms. Overall, NSM-FRP provided twice as much bond strength as EBR did. A higher ratio of vertical NSM-FRP provided an increase in shear strength of the wall panel, whereas a combination of horizontal and vertical NSM-FRP prevented opening of diagonal cracks and sliding failure during in plane shear loading. For out of plane flexural testing, it was noted that closer spacing of vertical NSM-FRP led to an increase in load capacity. Increasing the reinforcement ratio also led to improvement of load capacity but resulted in lower displacement.

NSM-FRP strips and bars also provided better fire resistance than EBR-FRP laminates. Smaller insulation length and thickness were required to prevent the adhesive from reaching its glass transition temperature. As long as the structure was well insulated, a two-hour fire rating could be achieved, even if the glass transition temperature was exceeded. Using a higher glass temperature epoxy and NSM FRP resulted in similar amounts of fire resistance as regular RC beams. Studies indicate that insulation of the anchorage zone is vital to improving structural performance, even when the FRP has lost its mechanical properties.

Future research should be focused on NSM-FRP shear strengthening design, as the current ACI 440 [1] requirements of edge clearance and spacing between adjacent NSM grooves can limit the shear strengthening applicability of NSM. Additionally, despite reducing FRP debonding failures, NSM strengthened specimens are prone to concrete cover splitting or fracture failures. To address both these drawbacks, experiments have shown that using hybrid composite plates (HCPs) or hybrid EBR plates bonded to the NSM FRP can be viable options. However, these methods still require further investigation. Although prestressing of NSM-FRP enhances the load carrying capacity performance, the ductility of the strengthened systems has shown a tendency to decrease. Preliminary experiments have tested partially bonded prestressed NSM-FRP systems and noticed a more balanced improvement of load capacity and ductility of strengthened specimens. None the less, there is a need for research on improving the ductility of the strengthened structures. Long term performance monitoring of concrete beams and slabs with NSM-FRP reinforcement is another possible area of research. Majority of the studies regarding strengthening using NSM FRP are performed on simply supported structural members. Investigations on continuously supported RC structures strengthened with NSM-FRP can provide some insight in this area of research as well. Finally, to improve the fire endurance of RC structures, the use of various bonding materials such as cementitious grouts and modified resins need to be explored in more details.

## Figures and Tables

**Table 1:** Summary of experimental work on the flexural strengthening applications of NSM FRP.

**Reference**	[7]	[8]	[9]	[10]	[11]
**Test type**	Flexure				
**Specimen**	Simply supported RC T-beams	Cantilevered RC bridge slab and RC beam	Simply supported RC beams	Simply supported RC beams	Simply supported RC beams
**Cross-section dimensions, *l* × *w* × *d* (mm)**	Span = 4,572, flange = 381 × 102, web = 152 × 305	Bridge spans = 61.1, 64.1, and 80.0, beam = 900 × 170 × 200	1,000 × 120 × 170	3010 × 200 × 300	2800 × 150 × 280
***f'*_c_** **(MPa)**	36.17	31.4	37.6–49.5	35	36.5–67.2
**Test method**	4 point bending	Cantilever, and three-point bending	Four-point bending	Four-point bending	Four-point bending
**FRP material type and configuration**	GFRP (deformed), and CFRP (sand blasted) rods	CFRP strips	CFRP sheet and laminate	CFRP, and GFRP rods	CFRP rods
**FRP diameter *d*_f_ or FRP sheet width *w*_f_ (mm) ^a^**	9.5 and 12.7	-	0.111–0.167	9.5, 12.7, 11.3 and 15.9	6 and 12
**FRP cross-section *h*_f_ × *t*_f_ (mm) ^a^**	-	16 × 2	10 × 1.4	-	-
**Groove dimensions *h*_g_ × *t*_g_ (mm) ^a^**	16 × 16, 19 × 19, and 25.4 × 25.4	20 × 4	N/A^b^	1.5–2 times d_f_^a^	12 × 12, and 24 × 24
**Test variables**	Bonded length, FRP rod diameter, material, and groove	Variable loading conditions, and long term durabilty test	Distance between FRP rods, and FRP reinforcement ratio	Steel reinforcement ratio, FRP rod diameters and bonded lengths, material	Concrete strength, and epoxy
**Observed failure mode**	Concrete crushing, FRP debonding	N/A ^b^	Delamination of concrete cover, FRP debonding and rupture	Steel yielding, and concrete cover splitting	Concrete cover peel-off, FRP pull-out and concrete crushing
**Increase in ultimate load**	25.7%–44.3%	41%	83% (averaged)	up to 104%	59.2–73.2 kN

**^a^** See Figure 1 for details; ^b^ N/A = Not available.

**Table 2:** Summary of experimental work on the shear strengthening applications of NSM FRP.

**Reference**	[13]	[14]	[15]	[16]
**Test type**	Shear			
**Specimen**	Simply-supported concrete and RC beams	Simply-supported RC beams	Simply-supported deep RC T-beams	Simply-supported RC beams
**Cross-section dimensions, *l* × *w* × *d* (mm)**	1500 × 150 × 300, and 900 × 150 × 150	2000 × 200 × 210	Span = 4200, flange = 450 × 100, web = 180 × 500	1650 × 200 × 250
***f'*_c_** **(MPa)**	37.6–49.5	29.3	40.1	36.4
**Test method**	Four-point bending	Four-point bending	Three-point bending	Three-point bending
**FRP material type and configuration**	CFRP strip	CFRP rods, and strips	CFRP rods	CFRP sheets wrapped around wooden rods
**FRP Diameter *d*_f_ (mm) ^a^**	-	7.5 and 8	-	-
**FRP Cross-Section *h*_f_ × *t*_f_ (mm) ^a^**	10 × 1.4	16 × 2	10 × 1.4, and 20 × 1.4	0.11
**Groove dimensions *h*_g_ × *t*_g_ (mm) ^a^**	12 × 5	15 × 15, and 18 × 5	N/A ^b^	15 × 15
**Test variables**	FRP spacing and inclination	FRP forms, epoxy, spacing and inclination of FRP	FRP dimensions, spacings and orientations	FRP end anchorage, spacings and orientations
**Observed failure mode**	Shear cracking	Shear cracking, and FRP debonding	Shear cracking, and FRP rupture	Shear-flexural cracking
**Increase in ultimate load**	83% (averaged)	22%–44%	66%–85%	25%–48%

**^a^** See Figure 1 for details; ^b^ N/A = Not available.

**Table 3:** Summary of experimental work on prestressing applications of NSM FRP.

**Reference**	[17]	[18]	[19]	[20]
**Test type**	Prestressing			
**Specimen**	Simply-supported RC beam	Simply-supported RC beam	Simply-supported RC beam	Simply supported RC slab
**Cross-section dimensions, *l* × *w* × *d* (mm)**	3300 × 300 × 350	3200 × 200 × 300	5150 × 200 × 400	2600 × 600 × 120
***f'*_c_** **(MPa)**	31	27	40	46.7
**Test method**	Three-point bending	Four-point bending	Four-point bending	Four-point bending
**FRP saterial sype and sonfiguration**	NSM CFRP laminate strips (sand coated)	NSM CFRP plates	NSM CFRP strips	NSM CFRP laminates
**FRP cross-Section *h*_f_ × *t*_f_ (mm) ^a^**	25 × 2	25 × 1.4	16 × 2	20 × 1.4
**Prestressing strain levels (%)**	5, 20 and 30	0–50	0, 20, 40, 60	20 and 40
**Test variables**	Prestressing levels under service-load state	Prestressing levels and transverse grooves	Fatigue, in-service loading conditions	Prestressing levels, under service and ultimate loads
**Observed failure mode**	FRP rupture	FRP debonding, and concrete cover seperation	FRP debonding and FRP slippage at end anchorages	FRP rupture
**Increase in ultimate load**	11.5%–15%	42%–96%	44.1–88.2 kN	136%–152%
**Reference**	[21]	[22]	[23]	[24]
**Test type**	Prestressing			
**Specimen**	Simply supported, RC slab	Simply supported, RC slab	Simply supported, stone slab	Simply supported, RC slab
**Cross-section dimensions, *l* × *w* × *d* (mm)**	2600 × 600 × 120	2400 × 150 × 300	3400 × 450 × 100	1800 × 150 × 300
***f'*_c_** **(MPa)**	15	32.2	142.4	27.7
**Test method**	4-Point bending	4-Point bending	4-Point bending	4-Point bending
**FRP material type and configuration**	NSM CFRP laminates	NSM CFRP laminates	NSM CFRP bars (rough surfaces)	NSM CFRP bars (spirally wound)
**FRP diameter *d*_f_ (mm) ^a^**	-	-	7	7.9
**FRP cross-section *h*_f_ × *t*_f_ (mm) ^a^**	20 × 1.4	20 × 1.4	-	-
**Groove dimensions *h*_g_ × *t*_g_ (mm) ^a^**	-	24 × 6	27 × 14	20 × 20
**Prestressing strain levels (%)**	20 and 40	0, 20, 30, and 40	0, 15 and 30	40
**Test variables**	Low concrete compressive strength, serviceability limit states	Prestressing levels, low steel reinforcement ratio of 0.39%	Prestressing levels, no steel reinforcement	Number of FRP bars, end treatments, and prestressing levels
**Observed failure mode**	FRP rupture	Concrete crushing and FRP rupture	FRP rupture, and excessive deflection	FRP bar slippage, and concrete crushing
**Increase in ultimate load**	125% and 134%	32%, 47%, 55% and 63%	30%–60% (Cracking load)	71%

**^a^** See Figure 1 for details.

**Table 4:** Summary of experimental work on masonry in plane shear strengthening applications of NSM FRP.

**Reference**	[33]	[34]	[35]		[36]	[37]
**Test type**	In plane shear					
**Specimen**	URM walls	URM walls	URM walls		URM walls	URM walls
**Wall dimensions, *h* × *l* (mm)**	1200 × 1200	1200 × 1200	1170 × 1170, and 1170 × 1075		1200 × 1200	1204 × 1190, and 1032 × 1910
**Masonry brick compressive strength *f'*_b_ (MPa)**	8.8–19.4	N/A ^b^	11.2–21.2		28.6	21.3
**Test method**	Diagonal tension	Diagonal tension	Diagonal compression		Cyclic shear and vertical compression	Cyclic shear
**FRP material type and configuration**	NSM CFRP strips	NSM CFRP strips	Anchored EB CFRP plates, and GFRP fabrics and strips	NSM CFRP strips	NSM CFRP strips	NSM CFRP strips
**FRP cross-section *h*_f_ × *t*_f_ (mm) or w (mm) ^a^**	12 × 1.2, 30 × 1.2	15 × 2.8	Width ranging from 50–150	15 × 1.2	10 × 1.4	15 × 2.8
**Groove dimensions *h*_g_ × *t*_g_ (mm) ^a^**	20 × 8, 35 × 8	20 × 6	N/A ^b^		N/A ^b^	15 × 8
**Orientation of FRP**	Vertical and diagonal	Vertical and horizontal	Vertical, horizontal and diagonal		Vertical and horizontal	Vertical and horizontal
**Test variables**	FRP orientation, number of strips, and position	FRP orientation, number of strips, and position	FRP form, orientation, number of strips, and position		FRP orientation, pre-compression levels, number of strips, and position	FRP orientation, and boundary conditions
**Observed failure mode**	Diagonal cracking, sliding and FRP debonding	Diagonal cracking, sliding and FRP debonding	Diagonal cracking, sliding and FRP debonding		Diagonal cracking	Diagonal cracking, and sliding
**Increase in wall strength**	1.3–3.7 times	1%–46%	31%–325%		9.30%	1%–9%

**^a^** See Figure 1 for details; ^b^ N/A = Not available.

**Table 5:** Summary of experimental work on masonry out of plane flexural strengthening applications of NSM FRP.

**Reference**	[38]	[39]	[40]	[41]
**Test type**	Out of plane flexure			
**Specimen**	URM wall, and column	URM walls	URM walls	URM walls
**Wall dimensions, *h* × *l* (mm)**	2313 × 1070, and 1710 × 355	3000–4100 × 1150, and 2700–4000 × 1170 × 1250	3900 × 2800	1170 × 1250
**Masonry brick compressive strength *f'*_b_ (MPa)**	17	17.1–39.5	16.9–27.6	8.8–32
**Test method**	Three and four-point bending	Three and four-point bending	Monotonically increasing lateral pressure	Lateral pressure
**Material type and configuration**	NSM CFRP strips	NSM CFRP strips	NSM CFRP cable	NSM CFRP strips, and NSM twisted steel bars
**FRP diameter *d*_f_ (mm) ^a^**	-	-	5	-
**FRP cross-section *h*_f_ × *t*_f_ (mm) ^a^**	10 × 4.2, 10 × 3.6, 10 × 7.2, 7.5 × 4.8, and 5 × 4.8	15 × 1.2	-	15 × 1.2
**Groove dimensions *h*_g_ × *t*_g_ (mm) ^a^**	N/A ^b^	20 × 8	N/A ^b^	N/A ^b^
**Orientation of FRP**	Vertical	Vertical	Vertical and horizontal	Vertical
**Test variables**	FRP spacing, reinforcement ratio, and cyclic and pre-compression loadings	Wall dimensions, one-or two-sided FRP retrofit	Low FRP reinforcement ratios, pre-compression loads, various support conditions	In-situ and pre-compression loads
**Observed failure mode**	IC debonding	Displacement Induced (DI) debonding	Diagonal cracking, compressive crushing of masonry, FRP slip, and shear rupture	FRP debonding, and FRP pull-out
**Increase in wall strength**	up to 20 times	3.05–6.21 times	25%–291%	440%–830%

**^a^** See Figure 1 for details; ^b^ N/A = Not available.

**Table 6:** Summary of experimental work related to NSM-FRP reinforced members under temperature.

**Reference**	[43]		[44,45]	[4]	[46]
**Test type**	Temperature				
**specimen**	N/A		Simply-supported RC beam	Simply-supported RC beam	Simply-supported RC beam
**Cross-section dimensions*l* × *w* × *d* (mm)**	N/A		3150 × 200 × 300	1524 × 254 × 102	4100 × 200 × 450
**Temperature range (°C)**	20–600		ISO 834	21–200	ISO 834
**Insulation type**	N/A^b^		Glass-fiber cement, CS ^c^, two component system and ceramic	Ceramic	Rock-wool and CS ^c^
**Insulation length (mm)**	N/A ^b^		2900	FRP bonded length	225–850
**Test method**	Tension test		Service load and EN 1363-1 standard fire	Monotonic or service load, and transient high temperature	Four-point bending, and standard fire
**FRP Material type and configuration**	CFRP strips and rods		CFRP and GFRP rods	CFRP fabric and tape	Basalt FRP bars
**FRP *T*_g_ (°C)**	80		82	59 and 122	N/A ^b^
**FRP diameter *d*_f_ (mm) ^a^**	6.4		9.5–16	-	10
**FRP cross-section *h*_f_ × *t*_f_ (mm) ^a^**	13.5 × 4.5		-	235 × 0.381, 16 × 2	-
**Adhesive or resin type**	High strength epoxy and expansive cement		Epoxy	Epoxy and cementitious material	Vinyl, heat resistance vinyl, and epoxy
**Adhesive *T*_g_ (°C)**	N/A ^b^		62–65	59 and 69	127, 174, and 210
**Groove dimensions *h*_g_ × *t*_g_ (mm) ^a^**	N/A ^b^		N/A ^b^	21 × 5	20 × 20
**Test variables**	High temperature, various epoxy and FRP forms		Various insulation systems	High temperature, various adhesive, and insulation lengths	High Tg resin, and insulation thickness
**Observed failure mode (s)**	Splitting or rupture of FRP fibers, resin decomposition		FRP debonding, and concrete crushing	FRP debonding	Flexural
**Thermal performance**	N/A ^b^		1–2 h	4 h at 100 °C, and 70 min at 200 °C	88–147 min
**Reference**	[47]	[48]	[49]	[50]	[51]
**Test type**	Temperature				
**Specimen**	Simply-supported RC beam	Simply-supported RC beam	Simply-supported RC T-beams	RC Concrete blocks	Simply-supported RC beam
**Cross-section dimensions *l* × *w* × *d* (mm)**	2100 × 100 × 120	1350 × 120 × 200	Span = 3962, flange 432 × 127, web = 229 × 279	350 × 120 × 120	1450 × 150 × 150
**Temperature range (°C)**	ISO 834	ISO 834	ASTM E119	20–150	N/A ^b^
**Insulation type**	CS ^c^, and VP ^d^ mortar	CS ^c^	U-shaped Tyfo^®^CFP ^e^	-	N/A ^b^
**Insulation length (mm)**	Anchorage zones	Anchorage zones	3180	-	Anchorage zones
**Test method**	Four-point bending, and standard fire	Four-point bending, and standard fire	Four-point bending, and standard fire	Double-lap shear test, and high temperature	DMA ^f^, TGA ^g^ and thermal conductivity, and four-point bending test, and thermal load
**FRP material type and configuration**	CFRP laminate	CFRP strips	CFRP strips	CFRP strips	CFRP bar
**FRP *T*_g_ (°C)**	N/A ^b^	83	N/A ^b^	83	220
**FRP diameter *d*_f_ (mm) ^a^**	-	-	-	-	N/A ^b^
**FRP cross-section *h*_f_ × *t*_f_ (mm) ^a^**	50 × 1.2	10 × 1.2	13.5 × 4.5	10 × 1.2	-
**Adhesive or resin type**	Epoxy	Epoxy, and mixed grout	Epoxy	Epoxy, and mixed grout	Cementitious grout
**Adhesive *T*_g_ (°C)**	55	47 and 44	N/A ^b^	47 and 44	N/A ^b^
**Groove dimensions *h*_g_ × *t*_g_ (mm) ^a^**	N/A ^b^	N/A ^b^	N/A ^b^	15 × 5	N/A ^b^
**Test variables**	Fire protection schemes, and Tg of epoxy	Adhesive type, and fire protection schemes	Fire protection zones, and schemes	Adhesive type, bonded length	Material testing procedures, and fire protection schemes
**Observed failure mode (s)**	Structural failure	FRP debonding	FRP decomposition	Shear failure, and adhesive failure	FRP debonding
**Thermal performance**	60–90 min	114 min	3 h	N/A	90 min

**^a^** See Figure 1 for details; ^b^ N/A = Not available; ^c^ Calcium cilicate; ^d^ Vermiculite/perlite; ^e^ CFP = Composite fire protection; ^f^ DMA = Dynamic mechanical analysis; ^g^ TGA = Thermogravimetric analysis.

## Abbreviations

The following abbreviations are used in this manuscript:

NSM: near surface mounting
EBR: externally bonding reinforcement
FRP: fiber reinforced polymers
CFRP: carbon fiber reinforced polymers
RC: reinforced concrete
ACI: American Concrete Institute
FIB: Fédération Internationale du Béton
CSA: Canadian Standards Association
HCP: hybrid composite plate
SHCC: strain hardening cementitious composite
URM: unreinforced masonry
ASTM: American Society of the International Association for Testing and Materials
ISO: International Organization for Standardization
IC: intermediate crack
BFRP: basalt fiber reinforced polymers
*T*_g_: glass transition temperature
*T*_d_: decomposition temperature
CS: calcium silicate
VP: vermiculite/perlite
MG: cement and epoxy binder mixed grout
DMA: dynamic mechanical analysis
TGA: thermogravimetric analysis
ETS: embedded through section
